# Supplementary material for: Characterization of BoHV-1 gG-/tk-/gE- Mutant in Differential Protein Expression, Virulence, and Immunity
Source: Vet Sci. 2021 Oct 27;8(11):253. doi: 10.3390/vetsci8110253 (PMC8621285; doi:10.3390/vetsci8110253)
Supplement: Supplementary file 1 [file vetsci-08-00253-s001.zip › vetsci-1352317-supplementary.pdf]

veterinary sciences

MDPI

Article

Characterization of BoHV-1 gG-/tk-/gE- Mutant in Differential Protein Expression, Virulence, and Immunity

Marawan Adel Marawan , Mingliang Deng, Chen Wang, Yingyu Chen, Changmin Hu, Jianguo Chen, Xi Chen, Huanchun Chen and Aizhen Guo

Supplementary Materials

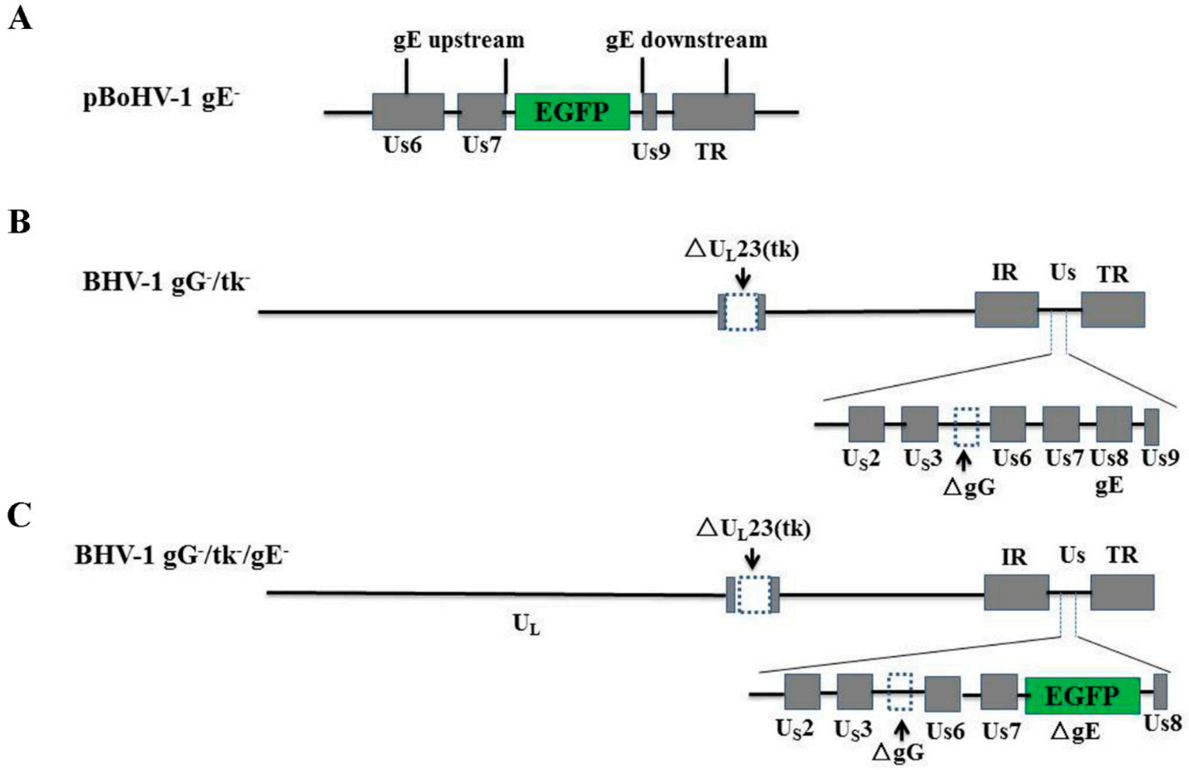

Figure S1. Construction strategy of pBoHV-1 gE^- and recombinant BoHV-1 $gG^-/tk^-/gE^-$ viruses based on BoHV-1 gG^-/tk^- mutant. (**A**) Construction of transfer vector, including target deletion region, upstream, downstream fragments and inserted EGFP expression cassette. (**B**) BoHV-1 gG^-/tk^- genome. (**C**) BoHV-1 $gG^-/tk^-/gE^-$ genome. The gE gene was deleted by insertion of EGFP expression cassette.

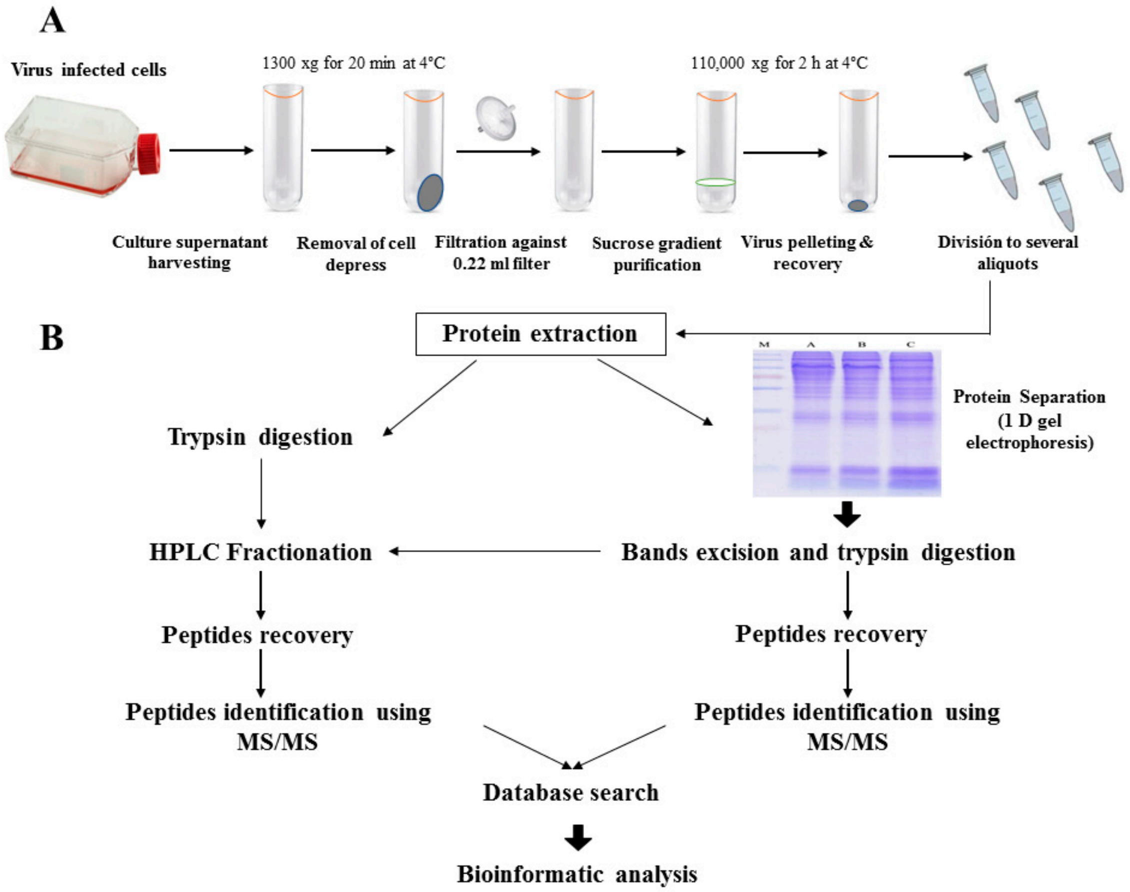

Figure S2. General workflow for the purification of the 4 different virions of BoHV-1 and mass spectrometry steps of LC.MS/MS**.** (**A**) Extracellular virions were purified using centrifugation/ultracentrifugation method on sucrose density gradient cushion. As the step of purity of crucial importance, EM observation is carried out to confirm the purity of the virions and absence of cell debris. Contaminating proteins were removed by the proteolysis of purified virions (**B**) Fig Analysis of virion-associated protein composition by mass spectrometry. Typical steps for analyzing virion-associated proteins by LC-MS/MS of extracted proteins of purified virions and separated proteins of purified virions using 1 D gel electrophoresis. Finally, database search and bioinformatic analysis were performed.

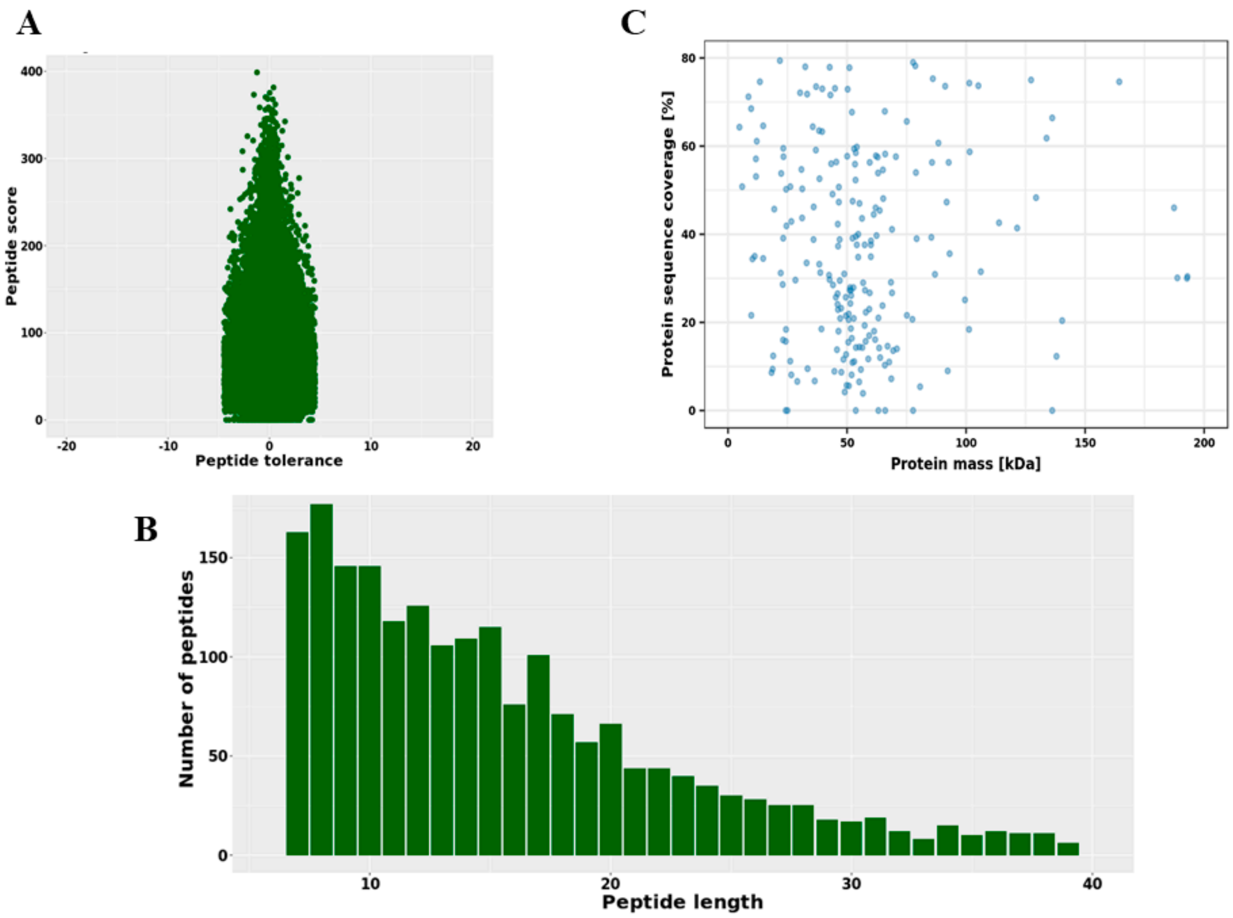

Figure S3. Quality Control (QC) Validation of MS Data**;** (**A**) Peptide mass tolerance distribution (**B**) identified peptide length distribution (**C**) protein mass and coverage distribution.

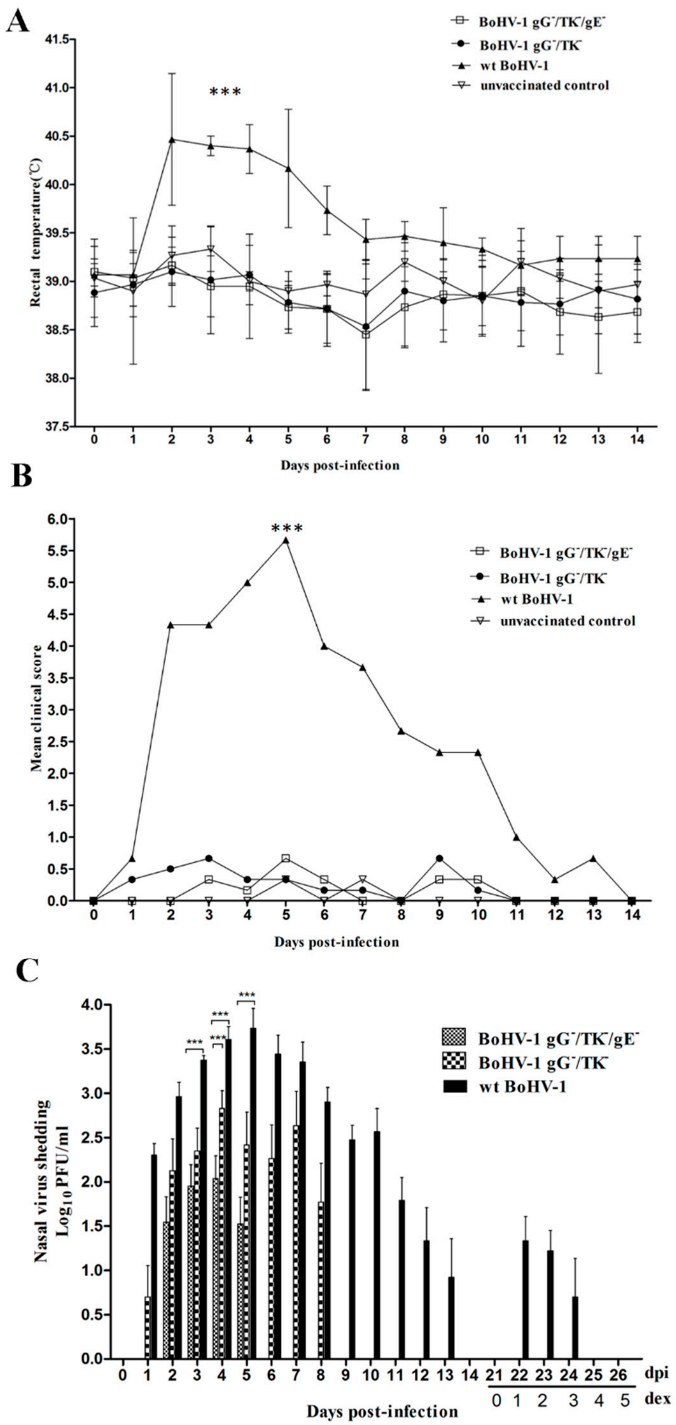

Figure S4. Clinical signs and reactivation of BoHV-1 mutant in calves. Temperature change (**A**), Mean daily clinical score (**B**), and nasal virus shedding (**C**) in calves following vaccination with BoHV-1 gG-/tk-/gE-, BoHV-1 gG-/tk- and wt BoHV-1 and after dexamethasone injection. Clinical signs combining body temperature, behavior, presence of coughs, abnormal respiration, ocular and nasal discharges, hyperemia, or lesions of the nasal mucosa, and conjunctivitis were scored as described earlier [1]**.** Nasal virus shedding was detected as described below: nasal swabs were submitted for virus isolation and quantitation. The nasal swab medium was filtered through 0.22 μm filters before being inoculated into MDBK cell cultures in 24-well tissue culture plates. The plates were monitored daily for characteristic BoHV cytopathic effects (CPE) for up to 4 days. The infectivity of the samples positive to the virus was subsequently quantified by plaque assay on MDBK cells in 24-well cell culture plates as described previously [2]**.**

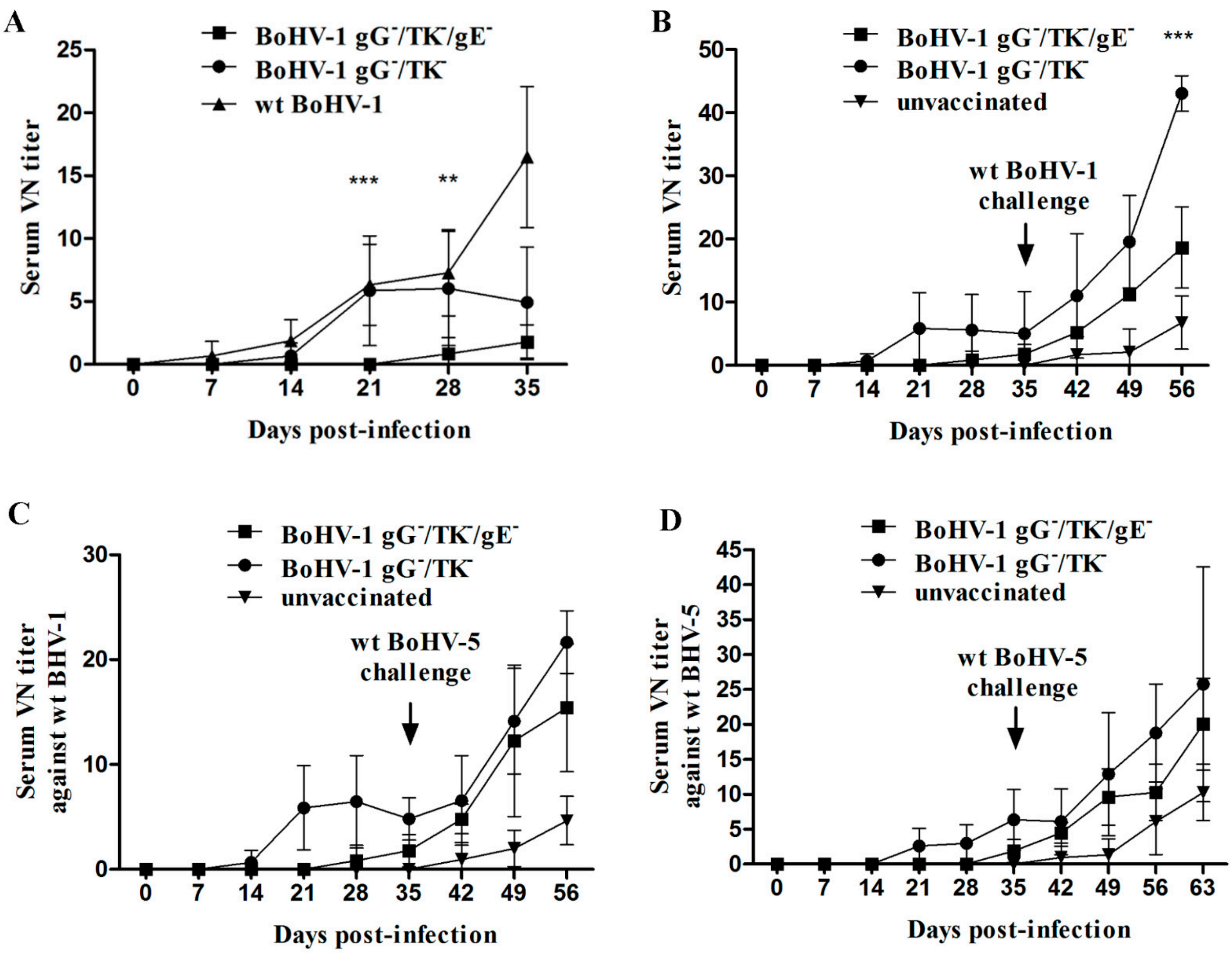

Figure S5. Virus-neutralizing (VN) antibodies titers in calves. Virus-neutralizing (VN) antibodies against BoHV-1 following vaccination (**A**) and challenge with wt BoHV-1 (**B**) or wt BoHV-5 (**C**). VN antibodies against BoHV-5 after challenge with wt BoHV-5 (**D**). VN antibodies were examined in a varying serum-constant virus neutralization (SN) assay in 96-well plates, with two-fold dilutions of serum against 100 TCID^{50} of either BoHV-1 strain HB06 or BoHV-5 strain EVI 88/95. The VN titers were expressed as the reciprocal of the highest serum dilution that prevented the development of cytopathic effect (CPE) after 72 h of incubation at 37 °C. The mark (**) with $p < 0.01$ and (***) with $p < 0.001$ were considered to indicate a significant or high significant statistical difference respectively.

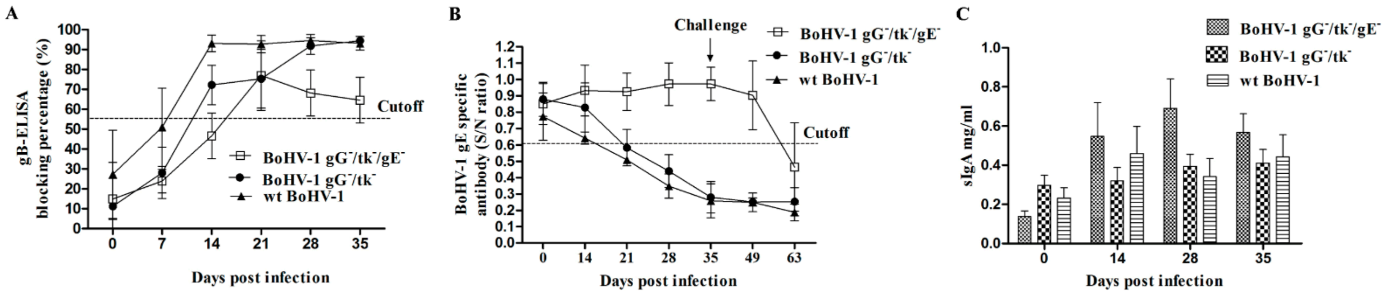

Figure S6. Antibody ELISA assays. Anti-BoHV-1 gB antibody (**A**), gE antibody (**B**) and serum sIgA (**C**) were determined in serum from vaccinated animals at different days post-infection. Serum samples with blocking % of 55% and greater are considered positive for BoHV-1 gB antibodies. Serum samples with an S/N ratio of 0.60 and lower are considered positive for BoHV-1 gE antibodies.

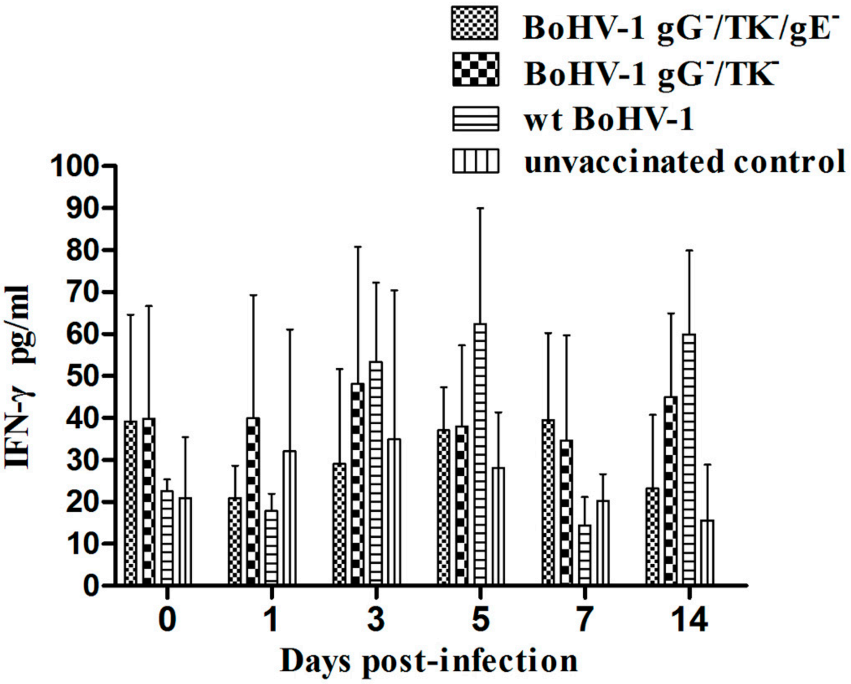

Figure S7. IFN-γ detection after vaccination.

Table S1. The primers used in this study.

Purpose	Primes and sequences 5′→3′[a]	Restriction sites	GenBank accession no	Position in the genome (nt)
gE upstream sequence	P1:CGAAAGCTTTCGCCTCCTGCCCGCG	*Hind*III	AJ004801	120554-120570
	P2:CTTTTGGTACCCTCTCGCGTGCGC	*Kpn*I	AJ004801	121694-121679
gE downstream sequence	P3:TCAAGTGGATCCTCCGCTAGGCG	*Bam*HI	AJ004801	123430-123444
	P4:CGAATTCCAAGCGCCGCCAGCGAG	*Eco*RI	AJ004801	124570-124553
EGFP gene	P5:GAACTGAGGTACCTACAGCGTGAGC	*Kpn*I	U55763	4396-4420
	P6:CGCGTTAGGATCCATTGATGAGTTTGG	*Bam*HI	U55763	1645-1619
Identification of gE gene	P7:GGGAGCGGGCAAGGCGGAGGAAGAC		AJ004801	121499-121523
	P8:GGCGCGTCCTCGATGGTGAGCTCGA		AJ004801	123657-123681
	P9:CCTGTTGCTCTGGATGGTGGTGCTG		AJ004801	120295-120319
	P10:GAATCAGGGGATAACGCAGGAAAGAACA		U55763	4676-4703
Identification of gG gene	P11: CCGACCGCCTCCTACACCAGATGCT		AJ004801	117102-117126
	P12: GGGTGTAGGCAAGCTCACCGCAACG		AJ004801	118937-118961
Identification of tk gene	P13:ACGGGCTGGGAAAGACAACAACGG		AJ004801	63315-63338
	P14:GCGGACACGTCCAGCACGAACA		AJ004801	64161-64182

a: The underlined sequence indicates the restriction sit.

Table S2. The total identified virion proteins of the three strains under investigation after LC-MS/MS peptides identification and database search using UNIPROT.

Protein accession No	Gene Name	Protein Description	Protein Metabolic Function and biological process	MS/MS Count wt BoHV-1	MS/MS Count Goat BoHV-1	MS/MS Count BoHV-1 gG-/tk-	MS/MS Count BoHV-1 gG-/tk-/gE-
11 Enveloped proteins							
O39506	US8	Envelope glycoprotein E	Cell-to-cell spread	155	172	186	0
O39504	US4	Envelope glycoprotein G	Cell-to-cell spread	185	158	1	3
O39498	UL1	Envelope glycoprotein L	Cell entry/cell-to-cell spread	43	30	35	28
K4P1A7	UL10	Envelope glycoprotein M	Cell entry/cell-to-cell spread	278	261	252	245
K4PEP2	UL22	Envelope glycoprotein H	Cell entry/cell-to-cell spread	199	193	176	145
K4P1I3	UL27	Envelope glycoprotein B	Cell entry/cell-to-cell spread	947	728	906	1152
Q77CE0	UL44	Envelope glycoprotein C	Cell attachment	646	420	355	666
K4P170	UL49.5	Envelope glycoprotein N	Unknown	94	75	75	87
K4P3M5	UL53	Envelope glycoprotein K	Cell entry/cell-to-cell spread	22	20	26	13
K4P3S4	US6	Envelope glycoprotein D	Cell attachment	337	242	211	322
O39505	US7	Envelope glycoprotein I	Cell-to-cell spread	146	129	136	37
8 Capsid proteins							
K4P3M0	UL6	Capsid portal protein	DNA encapsulation	56	62	62	66
K4P1J1	UL18	Capsid Triplex Subunit 2	Capsid morphogenesis	188	201	181	250
K4P3Q3	UL19	Major Capsid protein	Capsid morphogenesis	718	940	871	1036
K4PEN7	UL26	Capsid scaffolding protein	Scaffold/serine protease	56	76	75	87
Q77CD3	UL35	Small capsomere-interacting protein	Unknown	152	165	187	209
Q77CD6	UL38	Capsid Triplex Subunit 1	Capsid morphogenesis	147	170	142	194
K4P3L1	UL17	Capsid vertex component 1	Viral entry/release, viral genome packaging	107	115	96	77
O39492	UL25	Capsid vertex component 2	Viral entry/release, viral genome packaging	138	190	180	143
15 Tegument and nuclear egress proteins							
K4P3H9	Circ	Myristylated tegument protein		112	136	156	64
Q77CA9	UL3.5	Protein V57	Cell-to-cell spread	100	85	48	67
K4P1A3	UL16	Cytoplasmic envelopment protein 2	Possible virion egress &morphogenesis	97	112	123	95

K4P3L1	UL17	Capsid vertex component 1	Viral entry/release, viral genome packaging	107	115	96	77
K4P3K8	UL21	Tegument protein	Virion morphogenesis	110	143	158	95
O39492	UL25	Capsid vertex component 2	Unknown	138	190	180	143
K4PEN0	UL36	Very large tegument protein	Capsid transport	433	466	507	326
K4P1H6	UL37	Tegument protein	Virion morphogenesis	167	182	211	142
K4PEN4	UL31	Nuclear egress protein 1	Nuclear egress	20	44	27	24
Q77CD2	UL34	Nuclear egress protein 2	Nuclear egress	7	7	9	5
K4P3N0	UL49	Tegument protein VP22	Virion morphogenesis	398	368	321	238
O39501	US1.67	US1.67 protein	Unknown	54	34	16	32
Q77CC2	UL20	Virion protein	Unknown	22	20	26	11
Q77CD9	UL43	Virion protein	Unknown	61	42	46	50
O39507	US9	Virion protein	Anterograde transport (reactivation of latency)	4	7	3	0
31 Regulatory, Enzymatic and Cytoplasmic egress proteins							
Q77CC4	UL23	Thymidine kinase TK	DNA biosynthesis & latency reactivation	18	15	0	2
Q77CC6	UL28	Tripartite terminase subunit 1	Viral packaging and release	8	12	11	10
K4P189	UL29	Major DNA-binding protein	DNA replication	47	66	83	59
K4P3K0	UL30	DNA polymerase	DNA replication	27	61	30	24
K4P1H9	UL32	Packaging protein	Viral packaging and release	5	9	9	5
Q77CD1	UL33	Tripartite terminase subunit 2	Viral packaging and release	5	5	5	3
K4P179	UL39	Ribonucleoside-diphosphate reductase 1.	DNA replication	48	56	49	45
K4P3J2	UL40	Ribonucleoside-diphosphate reductase 2.	DNA replication	18	16	19	16
K4PEM5	UL41	Virion host shutoff protein,	Cellular mRNA degradation	159	155	157	40
K4P1H2	UL42	DNA polymerase processivity subunit	DNA replication	53	97	72	47
K4P3I7	UL46	Tegument protein VP11/12	Possible gene regulation	384	425	344	118
K4PEM0	UL47	Tegument protein VP8	Possible gene regulation	1045	894	428	808
K4P1G7	UL48	Trans-inducing factor bTIF/VP16	Gene regulation/virion morphogenesis	231	172	117	147
Q77CE6	UL51	Virion protein	Unknown	68	51	51	40

K4P1G4	UL52	DNA primase	Viral genome replication	22	25	22	5
K4P165	UL54	Multifunctional regulator, bICP27	Gene regulation; RNA metabolism	45	54	42	34
O39503	US3	Virion serine/threonine protein kinase	Unknown	105	126	5	70
K4P3M7	BICP0	Immediate-early transactivator protein	Regulation of cellular process	11	11	2	3
K4P1C2	BICP4	Immediate-early transactivator protein	Transcriptional regulator	262	357	132	91
K4P3M4	UL2	Uracil-DNA glycosylase	DNA repair	21	27	17	19
K4PEQ8	UL3	Phosphoprotein	Unknown	34	29	19	26
K4P1B2	UL5	DNA replication helicase	DNA replication	18	33	20	4
K4PEQ3	UL7	Cytoplasmic envelopment protein 1	Possible virion egress &morphogenesis	25	23	23	13
K4P1J6	UL8	DNA helicase/primase complex-	Viral genome replication	9	12	5	5
K4P3R1	UL9	Replication origin binding protein	DNA replication	10	22	12	9
K4PEP9	UL12	Alkaline nuclease	Metabolic process through endo & exonuclease activities	35	49	34	39
K4P1J4	UL13	Virion serine/threonine protein kinase	Serine/threonine kinase	18	22	22	27
Q77CB6	UL14	UL 14 protein	Viral process	36	30	29	15
K4PEP5	UL15	Tripartite terminase subunit 3	Viral packaging and release	18	27	17	13
K4P1D2	BICP22	Immediate-early and late transrepressor protein.	Unknown	52	58	65	39
Q77CE5	UL50	Deoxyuridine 5'-triphosphate nucleotidohydrolase	dUTP metabolic process	37	38	24	21

Table S3. Comparative upregulated and downregulated proteins detected in wt BoHV-1, double mutant BoHV-1 gG-/tk- and the triple mutant BoHV-1 gG-/tk-/gE-.

Protein accession	Protein description	A/C Ratio	Regulated Type	Function
	Wt BoHV-1 Vs double mutant BoHV-1 gG-/tk-			
Q77CC4	Thymidine kinase	7.832	Up	(Virulence + latency + immune evasion)
O39504	Glycoprotein G	1549	Up	(Virulence) + immune suppression
K4P3S4	Glycoprotein D	2.464	Up	(Virulence & Entry & targeted ab + T cells)
O39498	Glycoprotein L	1.507	Up	Cell entry/cell-to-cell spread
Q77CE0	Glycoprotein C	3.138	Up	(Virulence & Entry & targeted ab + T cells)
K4P170	Glycoprotein N	2.513	Up	Virulence and immunosuppression
K4P1A7	Glycoprotein M	1.626	Up	Virulence & cell to cell spread
K4P1C2	BICP 4	3.493	Up	Transcriptional regulator
K4P1G7	Alpha-TIF protein	2.686	Up	Gene regulation/virion morphogenesis
K4P3I7	UL 46 protein	1.605	Up	Possible gene regulation

K4P3R5	UL4 protein	3.061	Up	Unknown
Q77CE5	UL 50 protein	2.564	Up	dUTP metabolic process
K4PEM0	UL47 Vp8 protein	4.731	Up	Critical for viral replication and immunosuppression.)
K4PEM5	UL 41 protein	1.526	Up	Cellular mRNA degradation
K4PEP5	UL 15 protein	1.569	Up	Viral packaging and release
K4PEQ8	UL 3 Phosphoprotein	2.804	Up	Unknown
Q77CD9	UL 43 protein	3.161	Up	Virion entry
O39501	US1.67 protein	6.832	Up	Unknown
Q77CE6	UL 51 protein	2.384	Up	Virulence and viral growth
Q77CA9	UL3.5 protein	5.404	Up	Cell-to-cell spread
Q77CB6	UL14 protein	1.735	Up	Viral pocess
K4P189	Major DNA-binding protein	0.499	Down	DNA replication
Wt BoHV-1 Vs triple mutant BoHV-1 gG-/tk-/gE-				
Q77CC4	Thymidine kinase	8.882	Up	(Virulence + latency + immune evasion)
O39504	Glycoprotein G	1032.66	Up	(Virulence) + immune suppression
O39506	Glycoprotein E	740.2	Up	(Virulence) (Entry) (targeted ab) (reactivation)
O39505	Glycoprotein I	16.444	Up	Cell-to-cell spread
K4P3M5	Glycoprotein K	1.969	Up	(Entry, spread & infectious virus production)
K4P1B2	DNA replication helicase	3.037	Up	DNA replication
K4P1C2	BICP 4	2.863	Up	Transcriptional regulator
K4P1G4	DNA primase	2.733	Up	DNA replication
K4P3I7	UL 46 protein	3.669	Up	Possible gene regulation
K4PEM5	UL 41 protein	5.236	Up	Cellular mRNA degradation
K4PEQ3	UL 7 protein	2.334	Up	Possible virion egress &morphogenesis
O39501	US1.67 protein	1.528	Up	Unknown
Q77CB6	UL14 protein	1.886	Up	Viral process
Q77CC2	UL 20 protein	1.82	Up	Unknown
Q77CD9	UL 43 protein	1.691	Up	Virion entry
Q77CE6	UL 51 protein	2.473	Up	Virulence and viral growth
K4P1I3	Glycoprotein B	0.631	Down	(Virulence & Entry & targeted ab)
K4P3S4	Glycoprotein D	0.635	Down	(Virulence & Entry & targeted ab + T cells)
Q77CD6	Triplex capsid protein 1	0.526	Down	Capsid morphogenesis
K4P1J1	Triplex capsid protein 2	0.574	Down	Capsid morphogenesis
K4P1J4	US3 virion serine/threonine protein kinase	0.603	Down	Serine/threonine kinase
K4P3M0	Portal protein UL6	0.549	Down	DNA encapsidation
K4P3Q3	Major capsid protein	0.511	Down	Capsid morphogenesis
K4PEN4	Nuclear egress protein 1	0.665	Down	Nuclear egress
K4PEN7	Capsid scaffolding protein	0.43	Down	Scaffold/serine protease
K4PEP9	Alkaline nuclease	0.442	Down	Metabolic process through endo&exonuclease activities
Q77CC5	UL 24 protein	0.509	Down	Unknown
Q77CD3	Small capsomere-interacting protein	0.469	Down	Capsid morphogenesis
Double mutant BoHV-1 gG-/tk- Vs triple mutant BoHV-1 gG-/tk-/gE-				
K4P3M5	Glycoprotein K	1.612	Up	(Entry, spread & infectious virus production)
O39505	Glycoprotein I	13.254	Up	Cell-to-cell spread
O39506	Glycoprotein E	804.8	Up	(Virulence) (Entry) (targeted ab) (reactivation)
K4P1B2	DNA replication helicase	2.489	Up	DNA replication
K4P1G4	DNA primase	2.443	Up	DNA replication
K4P3H9	Circ protein	1.549	Up	Unknown

K4P3I7	UL 46 protein	2.286	Up	Possible gene regulation
K4PEM5	Virion host shutoff factor	3.431	Up	Cellular mRNA degradation
Q77CC2	UL 20 protein	2.279	Up	Unknown
Q77CD2	Nuclear egress protein 2	1.594	Up	Nuclear egress
K4PEQ3	Cytoplasmic envelopment protein 1	1.691	Up	Possible virion egress &morphogenesis
K4P170	Glycoprotein N	0.391	Down	Virulence and immunosupression
K4P1A7	Glycoprotein M	0.554	Down	Virulence & cell to cell spread
K4P1I3	Glycoprotein B	0.475	Down	(Virulence & Entry & targeted ab)
K4P3S4	Glycoprotein D	0.258	Down	(Virulence & Entry & targeted ab + T cells)
O39498	Glycoprotein L	0.623	Down	Cell entry/cell-to-cell spread
Q77CE0	Glycoprotein C	0.273	Down	(Virulence & Entry & targeted ab + T cells)
Q77CD6	Triplex capsid protein 1	0.365	Down	Capsid morphogenesis
K4P1J1	Triplex capsid protein 2	0.47	Down	Capsid morphogenesis
K4P3M0	Portal protein	0.469	Down	DNA encapsidation
K4P3M4	Uracil-DNA glycosylase	0.615	Down	DNA repair
K4P3M7	BICP0	0.511	Down	Regulation of cellular process + Regulation of cellular process + immunosuppression
K4P3Q3	Major capsid protein	0.481	Down	Capsid morphogenesis
K4P3R5	UL 4 protein	0.285	Down	unknown
K4PEM0	UL 47 Vp8 protein	0.26	Down	Critical for viral replication and immunosuppression.)
K4PEN7	Capsid scaffolding protein	0.456	Down	Scaffold/serine protease
K4PEP5	Tripartite terminase subunit 3	0.621	Down	Viral packaging and release
K4PEP9	Alkaline nuclease	0.381	Down	Metabolic process through endo&exonuclease activities
K4PEQ8	Phosphoprotein UL 3	0.258	Down	unknown
O39501	US1.67 protein	0.224	Down	unknown
Q77CA9	UL3.5 protein	0.261	Down	Cell-to-cell spread
Q77CD3	Small capsomere-interacting protein	0.446	Down	Capsid morphogenesis
Q77CD9	UL 43 protein	0.535	Down	Virion entry
Q77CE5	UL 50 protein	0.497	Down	dUTP metabolic process
K4P1G7	Alpha-TIF protein	0.402	Down	Gene regulation/virion morphogenesis

Table S4. Mean number of days of fever, ocular and nasal lesions, cough, and nasal virus shedding after intranasal inoculation.

Virus	Fever >39.7 °C	Nasal discharges	Ocular discharges	Cough	Virus shedding	Highest titer of virus shedding log_{10} PFU/ml (day)
BoHV-1 $gG^-/tk^-/gE^-$	0	0	0	0	3.5±0.8	2.04 (4)
BoHV-1 gG^-/tk^-	0	0.7±1.2	1.0±1.5	0	6.3±1.4	2.83 (4)
wt BoHV-1	4.7±1.5	3.3±2.0	8.0±1.0	3.7±4.7	12.0±1.0	3.73(5)
Unvaccinated control	0	0	0	0	0	0

Table S5. Mean number of days of fever, ocular and nasal lesions, cough, and nasal virus shedding after challenge.

Challenge virus	Virus	Fever > 39.7 °C	Nasal discharges	Ocular discharges	Cough	Virus shedding	Highest titer of virus shedding log_{10} PFU/ml (day)
wt BHV-1	BoHV-1 $gG^-/tk^-/gE^-$	1.3±0.6	0.7±0.6	3.7±4.0	0.3±0.6	9.3±1.1	4.18 (5)
	BoHV-1 gG^-/tk^-	0.7±1.1	0	2.7±3.8	0	6.0±1.0	3.18 (5)

	Unvaccinated control	3.3±0.6	2.7±0.6	8.0±2.6	1.0±1.7	11.3±1.5	5.32 (5)
	Blank control	0	0	0	0	0	0
wt BHV-5	BoHV-1 gG$^-$/tk$^-$/gE$^-$	0.7±1.1	0.3±0.6	4.7±1.2	0.3±0.6	9.3±1.5	3.25 (6)
	BoHV-1 gG$^-$/tk$^-$	0.7±0.6	4.0±2.6	2.3±1.5	0.3±0.6	8.0±1.0	2.88 (4)
	Unvaccinated control	3.0±2.6	2.0±1.7	6.3±3.2	1.7±1.5	12.0±1.7	4.06 (6)
	Blank control	0	0	0	0	0	0

Table S6. Assessment of protection rates based on different scoring systems.

Challenge virus	Scoring systems		Score means of the different groups			
			**BoHV-1 gG$^-$/tk$^-$/gE$^-$**	**BoHV-1 gG$^-$/tk$^-$**	**UC**	**NC**
wt BoHV-1	Clinical signs	Score means	0.82	0.50	2.0	0.07
		PE(%)	61.1	77.8	/	/
	Lung gross pathology	Score means	6.00	2.67	13.33	2.00
		PE(%)	64.7	94.1	/	/
	Total	Score means	6.82	3.17	15.33	2.07
		PE(%)	64.2	91.7	/	/
wt BoHV-5	Clinical signs	Score means	0.64	0.76	1.31	0.07
		PE(%)	53.8	44.2	/	/
	Lung gross pathology	Score means	6.00	9.00	15.33	2.00
		PE(%)	69.9	47.5	/	/
	Total	Score means	6.64	9.76	16.64	2.07
		PE(%)	68.6	47.22	/	/

Note: PE represents protection rate and was calculated according to the formula as follows:
PE% = (SUC − SNC) − (SVAC − SNC)/(SUC − SNC) × 100
= 1 − (SVAC − SNC)/(SUC − SNC) × 100
where S represents the score means of individual groups of UC (Unvaccinated control), NC (negative control), vaccination (VAC) groups BoHV-1 gG$^-$/tk$^-$/gE$^-$, and BoHV-1 gG$^-$/tk$^-$, respectively.

Table S7. IL-4 detection after vaccination. Number of IL-4 detectable animals (means ± SD).

**Group**	**Animals no**	**0 dpi**	**1 dpi**	**3 dpi**	**5 dpi**	7 dpi	14 dpi
		Positive animals (means ± SD) IU/μg					
BoHV-1 gG$^-$/tk$^-$/gE$^-$	6	1 (0.04±0.1)	1 (0.5±1.1)	2 (4.7±8.3)	2 (9.1±14.4)	2 (4.5±8.7)	1 (0.3±0.6)
BoHV-1 gG$^-$/tk$^-$	6	3 (2.0±3.3)	6 (14.8±12.9)	2 (4.9±11.3)	4 (9.4±12.1)	5 (14.5±13.3)	2 (56.0±135.259)[a]
wt BoHV-1	3	0	0	0	0	0	0

[a]: The letter represents the difference between groups. The level of IL-4 in BoHV-1 gG$^-$/tk$^-$ vaccinated group was higher than that of BoHV-1 gG$^-$/tk$^-$/gE$^-$ and wt BoHV-1 vaccinated group ($p < 0.05$).

Reference

1. Wei, H.; He, J.; Paulsen, D.B.; Chowdhury, S.I. Bovine herpesvirus type 1 (BHV-1) mutant lacking UL49.5 luminal domain residues 30–32 and cytoplasmic tail residues 80–96 induces more rapid onset of virus neutralizing antibody and cellular immune responses in calves than the wild-type strain Cooper. *Veter-Immunol. Immunopathol.* **2012**, *147*, 223–229, doi:10.1016/j.vetimm.2012.04.015.
2. Chowdhury, S.I. Construction and characterization of an attenuated bovine herpesvirus type 1 (BHV-1) recombinant virus. *Veter- Microbiol.* **1996**, *52*, 13–23, doi:10.1016/0378-1135(96)00043-0
